# Perspectives of older women in the Netherlands: identifying motivators and barriers for healthy lifestyles and determinants of healthy aging

**DOI:** 10.1186/s12889-023-15611-0

**Published:** 2023-04-11

**Authors:** L. D. Sialino, H. A.H. Wijnhoven, S. H. van Oostrom, H. S.J. Picavet, W. M.M. Verschuren, M. Visser, S. Vader, L. A. Schaap

**Affiliations:** 1 Department of Health Sciences, Faculty of Science, Amsterdam Public Health research institute, Vrije Universiteit Amsterdam, De Boelelaan 1105, North-Holland, 1081HV, Amsterdam, the Netherlands; 2 Centre for Nutrition, Prevention and Health Services, National Institute for Public Health and the Environment, Bilthoven, the Netherlands; 3 Julius Centre for Health Sciences and Primary Care, University Medical Centre, Utrecht, the Netherlands

**Keywords:** Healthy aging, Lifestyle, Motivators and barriers, Older people, Perspectives

## Abstract

**Background:**

Women have a higher life expectancy than men but experience more years with physical disabilities in daily life at older ages, especially women with a migration background. This pinpoints older women as an important target group for strategies that stimulate healthy lifestyle, which benefits healthy aging. Our study investigates motivators and barriers for healthy lifestyles and perspectives on determinants of healthy aging of older women. This provides essential information for developing targeted strategies.

**Methods:**

Data was collected by semi-structured digital interviews from February till June 2021. Women aged 55 years and older living in the Netherlands (n = 34) with a native Dutch (n = 24), Turkish (n = 6) or Moroccan (n = 4) migration background were included. Two main subjects were investigated: (1) motivators and barriers on their current lifestyles regarding smoking, alcohol consumption, physical activity, diet and sleep and (2) perspectives on determinants of healthy aging. Interviews were analyzed using Krueger’s framework.

**Results:**

Personal health was the most common motivator for a healthy lifestyle. In addition, peer pressure and being outdoors were specific motivators for physical activity. Bad weather conditions and personal dislike to be active were specific barriers. The social environment, personal preferences and personal belief to compensate with other healthy lifestyle behaviors were barriers for low alcohol consumption. Personal preferences (liking unhealthy food and not making time) were the main barriers for a healthy diet. Sleep was not perceived as a form of lifestyle behavior, but rather as a personal trait. Since there were no smokers, specific barriers were not mentioned. For Turkish-Dutch and Moroccan-Dutch women, additional barriers and motivators were culture and religion. These were strong motivators to abstain from alcohol consumption and smoking, but a barrier for a healthy diet. With regard to perspectives on determinants of healthy aging, positive views on aging and being physically active were perceived as most important. Women often wanted to increase their physical activity or healthy diet to stimulate healthy aging. Among Turkish-Dutch and Moroccan-Dutch women, healthy aging was also perceived as something in the hands of God.

**Conclusions:**

Although motivators and barriers for a healthy lifestyle and perspectives on healthy aging vary for distinct lifestyles, personal health is a common motivator across all lifestyles. Having a migration background added culture and religion as distinct barriers and motivations. Strategies to improve lifestyle among older women should therefore have a tailored, culture sensitive approach (if applicable) for distinct lifestyle factors.

## Introduction

The older the population, the higher the proportion of women compared to men. This phenomenon is also known as the “feminization of aging” [1]. In the Netherlands, women account for approximately 60% of those aged 80 years and older due to their higher life expectancy [2]. Although women live longer, they experience relative more years with physical disabilities in daily life [3]. Older Turkish-Dutch or Moroccan-Dutch women form an additional risk group for unhealthy aging, due to a higher prevalence of chronic diseases, mobility difficulties and earlier onset of physical decline compared to their male counterparts and native Dutch older women [4–6]. This “female disadvantage in healthy aging” pinpoints older women (especially older Turkish-Dutch and older Moroccan-Dutch women) as an important group to target with strategies that stimulate healthy lifestyle, as this benefits healthy aging up to very old age. This is essential from both a public health perspective (aging society) and the wishes of older women themselves [7, 8].

Healthy lifestyle is known to play an essential role in healthy aging [9]. Changes towards a healthier lifestyle, such as an increase in physical activity, cause less disabilities and a slower decline in physical health in older adults [10, 11]. However, identifying motivators and barriers for healthy lifestyle behavior and perceived determinants of healthy aging among *older* women specifically remains relatively unstudied. Among adults, motivators and barriers for several healthy lifestyles have been studied and demonstrate significant differences between women and men [12–14]. For example, barriers to stop smoking among women are stress related, while in men they are social environmental related [14]. Furthermore, several studies have shown that motivators and barriers for physical activity and a healthy diet differ between younger adults and older adults [15, 16] For example, the social environment was the most important motivator for physical activity among older adults (65 years and older), while among adults (aged 50 to 64 years) this was stronger related to achieving their own goals [15]. Of the five main lifestyle factors (smoking, alcohol consumption, physical activity, diet and sleep), motivators and barriers for physical activity and a healthy diet have been most frequently studied among older adults, as well as among older women specifically [17–19]. For example, it was demonstrated that motivators related to social relationships have a greater influence on older women compared to men (aged 65 years and older) [17]. However, the majority of studies researching motivators and barriers for a healthy lifestyle do not investigate and/or report their results for *older* adults in general and rarely *separate by sex.* [20]. Furthermore, most research investigates one main lifestyle factors or the general concept of a healthy lifestyle, while literature suggests differences occur in motivators and barriers for different lifestyle factors [21, 22].

The motivators and barriers for a healthy lifestyle among older adults with a migration background remain relatively unstudied, especially for older women. Only a few studies demonstrated differences in motivators and barriers for physical activity among older adults (65 years and older) between ethnic groups in America and Australia [23, 24], but these studies did not specifically focus on women or differentiated according to sex. A recent focus group study in the Netherlands demonstrated that Moroccan-Dutch women (aged 44 to 60 years) have a different perception of healthy lifestyles and health compared to older native Dutch women [25]. They view their health as a gift from God, whereas native Dutch older women view it more as an individual responsibility and as something they can control themselves [25]. This suggests that when investigating motivators and barriers for different lifestyle behaviors, migration background should be taken into account.

We performed a qualitative study in order to explore motivators and barriers for the five main healthy lifestyles separately and the perspectives of older women on determinants of healthy aging of older women in the Netherlands. This study includes older Native Dutch, Moroccan-Dutch and Turkish-Dutch women living in the Netherlands, taking migration background into account. This provides essential information for developing strategies that stimulate healthy lifestyle behavior among all older women.

## Materials and methods

### Participants

Women aged 55 years and older living in the Netherlands with a native Dutch, Turkish or Moroccan migration background were included in the sample. The latter are two large migration groups in the Netherlands and were therefore selected to be part of this study. Since migrant adults have an earlier onset of physical decline compared to non-migrant adults, an age range of 55 years and older was chosen to include older Dutch, Turkish-Dutch and Moroccan-Dutch women with (some) decline in physical functioning, while they still have (the most) years to gain from preventive strategies [26, 27]. Women across different age groups and educational levels were included. Data collection was continued until saturation was reached, meaning that rarely new information was gained by an additional interview [28]. In total 34 women were included with a mean age of 66 years (SD = 8 years), with twenty-four native-Dutch, six Turkish-Dutch and four Moroccan-Dutch older women.

### Data collection

The study draws on qualitative data collected by means of online semi-structured interviews held between February and June 2021. The study was non-WMO approved by the ethical committee of the Vrije Universiteit medical center (METC Amsterdam UMC, approval number 2020.0726). The participants were recruited via convenience sampling and purpose sampling using contact persons in community centers, mosques, volunteer organizations, cultural federations, medical associations and our existing network. Flyers with information about the interviews were distributed to eligible participants by the main researcher or via key persons in the corresponding organizations. Women who expressed their interest were contacted by the main researcher and an interview was scheduled. The participants received an introductory e-mail or phone call upfront. Due to the corona pandemic, the interviews were conducted via Zoom (online communication tool with video) or via (mobile) phone if participants were unable to use Zoom. All participants provided written or audio recorded informed consent. All interviews were audio recorded and subsequently transcribed. Before the start of the interview, the participants provided written or audio recorded informed consent. After the interview, each participant received a financial incentive (€20 voucher) and an information package on healthy aging. The average duration of the interview was 36 min (SD 10 min). Three interviews were performed without digital image due to the lack of computer or telephone skills by the participant.

### Interview guide

The interview guide entailed general questions, such as age, migration background, educational level (categorized into low (elementary education or less), middle (lower vocational education and general intermediate education) and high education (intermediate vocational education, general secondary education, higher vocational education, college education and university)), paid job and living situation. Thereafter, two main research objectives were questioned: (1) motivators and barriers for healthy lifestyle (smoking, alcohol consumption, physical activity, diet and sleep) and (2) perspectives on determinants of healthy aging. Motivators were defined as reasons to have a healthy lifestyle behavior and barriers were defined as reasons not to have a healthy lifestyle behavior, discussed for each distinct lifestyle.

### Analysis

Data analysis followed Krueger’s framework [29], which consisted of a number of interconnected steps: familiarization with the data; coding; and interpretation of existing links between codes. In more detail, a thematic content analyses based on grounded theory was performed for each interview, consisting of open, axial and selective coding using Atlas.ti 9 [29]. Grounded theory describes the development of the codes and themes from the material itself, rather than using predefined codes and themes in the analysis [30]. Two independent researchers discussed the analytical steps to avoid researcher bias. During data analysis, the codes and themes were discussed and evaluated within the research team to reach consensus (researcher triangulation). The codes of all interviews were checked again after the final themes were created by the main researcher to stimulate reliability. A conceptual framework was build based on the themes of motivators and barriers that emerged from analyzing the interview transcripts. The main motivators and barriers (most often mentioned) are discussed per lifestyle; smoking, alcohol consumption, physical activity, diet and sleep, including supporting quotes. Themed results and quotes were translated into English by the main researcher. If applicable, consistently emerging differences between age groups, educational levels and migration backgrounds are discussed.

## Results

### Characteristics interviewed women

In total 34 women were included with a mean age of 66 years (SD = 8 years) (Table 1). There were seventeen younger old women (55–65 years), eleven middle old (65–75 years) and six older old women (75 + years). Twenty-four native-Dutch, six Turkish-Dutch and four Moroccan-Dutch older women participated. Fourteen women had a paid job and seven lived alone.

**Table 1:** Characteristics of participants

Characteristics	Participants (n = 34)
Female (n)	34
Age in years (mean (SD))	65.9 (7.9)
55–65 (n)	17
65–75 (n)	11
75+ (n)	6
Migration background (n)	
Dutch	24
Turkish	6
Moroccan	4
Educational level * (n)	
Low	14
Medium	8
High	12
Paid job (n)	14
Living alone (n)	7

* Education was categorized into low (elementary education or less), middle (lower vocational education and general intermediate education) and high education (intermediate vocational education, general secondary education, higher vocational education, college education and university)

### Barriers and motivators for healthy lifestyles

**Fig. 1:**
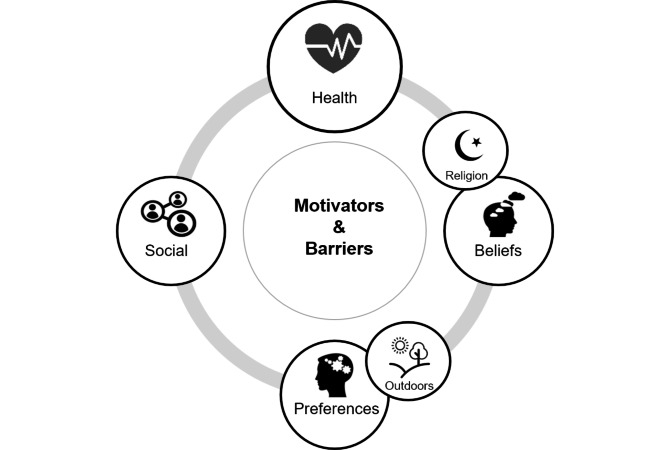
Schematic overview of conceptual framework of motivators and barriers of healthy lifestyle behavior. The framework is based on factors influencing the decision to engage in a certain lifestyle behavior that emerged from reviewing the interview transcripts. Four themes were identified: 1) Health (physical and mental health), 2) Beliefs (religion, views and personality characteristics) 3) Preferences (likes and dislikes, most often related to being outdoors and 4) Social environment (peer pressure, friends and family).

Four themes of barriers and motivators for healthy lifestyles were identified and formed our conceptual framework: health (physical and mental health), beliefs (mindset, religion and personality characteristics), preferences (what they like) and social environment (peer pressure, friends and family) (Fig. 1). The barriers and motivators for each healthy lifestyle are discussed in detail below.

#### Smoking

None of the women were current smoker and only few (n = 7) had smoked in the past (a long time ago). Turkish-Dutch and Moroccan-Dutch women often viewed not smoking part of their religion and cultural behavior. *“I do not smoke or drink ever, because I am Muslim.” – Turkish-Dutch woman, 65–75 years.* Common mentioned motivators among native-Dutch women were the known negative effects of smoking on their own health and wanting to set a good example for their children. *“I do not smoke because of my health. It also helped that I was pregnant and that helped me to quit smoking and thereafter I never started again. That helped, but above all it was my health.” – Native Dutch woman, 55–65 years.* Women with a history of smoking mentioned pregnancy as a motivator to stop smoking at that time. Since none of the women currently smoked, barriers to smoke less or abstain from smoking were not discussed.

#### Alcohol consumption

None of the Turkish-Dutch and Moroccan-Dutch women consumed alcohol. Of the native-Dutch women, about two-third of the women regularly consumed alcohol, ranging from some drinks during the weekend to a few drinks every day. The other one-third rarely consumed alcohol, ranging from never drinking alcohol at all to only drinking alcohol on special occasions. Turkish-Dutch and Moroccan-Dutch women often viewed abstaining from alcohol consumption part of their religion and cultural behavior. *“We do not drink as Muslims. So we are living healthy in that way.” –Turkish-Dutch woman, 55–65 years.* Most women who regularly consume alcohol viewed this as unhealthy behavior. Some said it was not *that* unhealthy, which was substantiated with arguments such as: they could have drank more excessively; others drink more excessively; or they believe to compensate with other healthy behavior. *“Yeah, I drink alcohol. I drink about two glasses every evening and that is maybe too much, but I never drink more than that. That is a healthy choice” – Native Dutch woman, 65–75 years. “I know it is unhealthy, but I really like it. So I allow myself to drink. For other stuff I am very healthy, I eat out of my own garden, so it is ok I think.” – Native Dutch woman, 55–65 years.* Other mentioned barriers for consuming no or low alcohol among native-Dutch women were liking the taste and/or the feeling when consuming alcohol and experiencing it as an important part of social activities such as parties and going out for dinner. *“When I am visiting someone, I want to drink a glass or when I am at dinner or something.” – Native Dutch woman, 65–75 years.* The main motivators to consume no or little alcohol were the known negative effects on their own health and disliking the taste and/or feeling at the moment of consumption or the next day (feeling more tired and less physically fit).

#### Physical activity

Almost all women performed several physical activities during the day. Most common physical activities were walking, biking, doing household work (cleaning and groceries), gardening and being physically active at work. Dutch younger and middle old women (until 75 years) also performed sports such as golf, swimming, tennis and/or going to the gym. Almost all viewed their physical activities as healthy, and more than half of these women mentioned they should and would like to be more active than they were at that moment. The most common motivators for being physically active were the known or experienced positive effects on their health and social peer pressure. In more detail, for physical health, prevention of high cholesterol and weight gain (most specific for Turkish-Dutch and Moroccan-Dutch women) and less joint and lower back pain, muscle stiffness and/or menopause complaints were mentioned. Also, positive effects on mental health were often mentioned, such as feeling relaxed. *“Because it is healthy and it (physical activity) makes me feel really good. I feel flexible and in shape. That is really motivating. It just feels good for my body and my mind.” – Native Dutch woman, 55–65 years.* Peer pressure as a motivator was described as having an appointment with a sports team, friends, family or walking the dog (specifically for native Dutch women). For some native-Dutch women a specific deal with themselves was also mentioned as a strong motivator, which seemed part of personal characteristic. “*I make the deal with myself that I do sports three times a week. If I then only go two hours, I can get cranky or feel guilty, and I do not like that.” – Native Dutch woman, 55–65 years.* Being outdoors in the fresh air and nature was a common motivator for all women. *“Yeah, I am always looking to connect with the outdoors. Getting some fresh air and talking to other women. That is how I stay in contact, I try to arrange walking together every day.” – Moroccan-Dutch woman, 55–65 years.* In line with this motivator, the most often mentioned barrier for physical activities was bad weather conditions. Other common barriers were having other plans i.e. no time (work, kids visiting or other social activities), not having someone to be physically active with (most common among women who lived alone) and not liking to be physically active (among relatively inactive women, most of which were Turkish-Dutch and Moroccan-Dutch women). In addition, feeling tired was a barrier specifically mentioned by Turkish-Dutch and Moroccan-Dutch women. Women who had a history of physical complaints, mentioned their fear to fall, get injured or overburden their body to be important barriers (especially among Turkish-Dutch and Moroccan-Dutch women). *“I just do not feel like it. I have pain and when you are old it is more difficult. I just do not feel like it often. You also have to be more careful” – Turkish-Dutch woman, 55–65 years.*

#### Diet

Most women described several aspects of their diet that hey considered healthy, such as a diet containing fruit and vegetables or containing fresh products, a diet being low in salt, sugar, meat, and fat and consisting of moderate-size portions. More than half of the women viewed their overall diet to be healthy, while others mentioned they could eat healthy more consistently healthy or healthier in general (especially Turkish-Dutch and Moroccan-Dutch women). *“I try to eat healthy, yes. Well, because you read a lot more about it than before. Sometimes there are days when I do not think about it and eat unhealthier such as sweets. I regret it the next day. I should be more consistent” - Native Dutch woman, 55–65 years.* Unhealthy aspects of their diet were eating sweets, fast food or high fat dishes. The most often mentioned motivator for consuming a healthy diet was the known beneficial effect on health, especially on cholesterol levels and feeling physically fit. Most women indicated that their understanding of what is healthy in a diet and its health benefits has increased over the years due to more attention and information via the news, magazines and their children. *“I try to eat healthy and not gain weight to stay healthy. My kids tell me so.” – Turkish-Dutch woman, 55–65 years.* Native Dutch women also mentioned physical appearance related to healthy weight as an important motivator. Most often mentioned barriers for a healthy diet were not liking the taste of healthy foods, not making or having the time to cook healthy, feeling obligated to eat unhealthy at social events and wanting to eat cultural (more unhealthy) dishes (specifically for Turkish-Dutch and Moroccan-Dutch women). *“We eat healthy at home, but I do miss our cultural dishes. They taste so good. Sometimes we get back into our unhealthy food habits, but we try to eat healthy” – Turkish-Dutch woman, 55–65 years.*

#### Sleep

Most women considered their sleep quality as good and some as poor. In more detail, poor sleep quality was interpreted as not enough or too much sleep (sleep duration) and sometimes as difficulty falling asleep or waking up too early or during the night (sleep quality). When discussed further, women rarely considered sleep a lifestyle behavior, but rather as a personal characteristic or a consequence of aging. *“I am a light sleeper and do not sleep long, that is part of me.” Native Dutch woman, 65–75 years*. The few women who considered sleep a lifestyle behavior mentioned ensuring enough sleep hours by going to bed early or not watching television before going to sleep. Their motivators for doing so were the known positive effect on health, such as positive effects on being physically fit and mental health. *“If you lay in bed 12 hours then you are stiff when you get out of bed, but if you lay for 8 hours I feel much more physically fit.” Native Dutch, 55–65 years.* Mentioned barriers for healthy sleep behavior were wanting to do more in a day and feeling stressed.

#### Perspectives on determinants of healthy aging

Most women had a positive view on their future aging. They explained to have a positive mind set and expect to or are currently able to cope with a decline in health and keep the focus on what you still can do. They mentioned this positive mindset and staying physically and mentally active to be most important for healthy aging in the future. *“I hope to age healthy, I aim to be very physically fit and active until I am 90 years old. That is what I hope. I try my best and hope it will work. But, you never know what might happen. You could have bad luck, but then just accept this. I do have the expectation to be a fit old woman.” – Native Dutch woman, 55–65 years.* Some women had a less optimistic view on future aging. They were worried about their physical decline or current physical complaints or did not like the idea of becoming older at all. *“With some fear and to say it plain, I do not want to become old and have disabilities at all.” – Native Dutch woman, 55–65 years.* Women with a negative view on healthy aging less often felt that they could influence the aging process with lifestyle changes. Few women never thought about future healthy aging process. They were most often younger and had no physical complaints, or did not experience a decline in physical functioning over the past years. *“I never really thought about it (aging), I cannot imagine it to be honest.” – Native Dutch woman, 55–65 years.* Turkish-Dutch and Moroccan-Dutch women described their future healthy aging to, in the end, be in the hands of Allah. *“I do not know how to answer that (what they saw as determinants of healthy aging), because it is in the hands of Allah.” – Moroccan-Dutch woman, 65–75 years.* A few mentioned that living healthy might help, but that aging is eventually beyond their control. *“I hope to age healthy, but I believe that it is up to Allah. I try to live healthy though.” – Turkish-Dutch woman, 65–75 years.*

## Discussion/conclusion

### Motivators and barriers for healthy lifestyles

A wide variety of motivators and barriers for a healthy lifestyle from the perspective of older women have been identified in our study and placed in a conceptual framework (Fig. 1). The most important motivator for a healthy lifestyle among older women in the Netherlands was the positive effect on personal health. This is in line with previous research demonstrating personal health as an important motivator to abstain from smoking among native Dutch adults [31], to be physically active among older native Dutch, Turkish-Dutch and Moroccan-Dutch older adults [32, 33], to eat healthy among native Dutch, Turkish-Dutch and Moroccan-Dutch (older) adults [32, 34] and sleeping enough hours among Dutch adults [35]. Although our results also demonstrated personal health as an important motivator for consuming no or little alcohol, this seems less often mentioned in literature. Rather, a strong skepticism towards the negative effects of alcohol on health has been found among older adults [36]. Since our participants are rather highly educated and specifically female, the knowledge on effects on health might be better understood and acknowledged. In line with previous research, the health of their unborn child was a motivator to not smoke during pregnancy [31, 32].

The second most important motivator for a healthy lifestyle were religious and personal beliefs. Among Turkish-Dutch and Moroccan-Dutch women, religious beliefs were the main reason to not consume alcohol or smoke (i.e. motivator). This is in line with very low rates of alcohol consumption and smoking among these women in the Netherlands [4, 37]. In contrast, among native Dutch older women, personal beliefs about alcohol consumption were a barrier to consume no or low alcohol. A general sense of downplaying the negative effect on health or compensating explanations was mentioned. The first is in line with a found downplaying of the negative effects of alcohol consumption on health [36] and the latter has been demonstrated for other unhealthy lifestyles. For example, the justification of smoking by a compensation effect of e.g. exercise and a healthy diet [38, 39]. Here, the risk is not denied, but rather reshaped by the person themselves based on an interplay with other lifestyle choices. This may also be related to the social culture surrounding alcohol consumption, as it is part of the Dutch culture to consume alcohol at social and work gatherings such as diners or birthdays [40].

Although also mentioned as barriers, the social environment and personal preferences were found to be common motivators for a healthy lifestyle regarding physical activity. These findings are in line with a recent review among older adults (65 years and older) demonstrating that personal health, social interactions and preference for being active were the main motivators for physical activity [41] and a recent study among native Dutch, Turkish-Dutch and Moroccan-Dutch adults demonstrating that peer pressure was a specific motivator for physical activity, especially among friends and family [32]. Commonly mentioned barriers for physical activity such as time restrains, personal dislike, fear of falling and injury and feeling tired have also been demonstrated in previous research among older adults (65 years and older) [32, 41]. Although previous research identified a financial barrier among lower educated adults [41], this was not found in our study. This might be because the majority of physical activities reported in our interviews were free of charge (walking and biking) while in other studies sports were a large part of physical activity. Also, relatively more women with an intermediate or high education level participated that who less likely experience a financial barrier.

Although not specifically mentioned as motivator or barrier, a personal positive view on future aging also seems to work as a motivator to for a healthy lifestyle among older women. Women who had a positive view often explained their lifestyle behavior as healthy. This is in line with previous research demonstrating that older adults, with a positive perspective on aging, tend to rate their lifestyle more often as healthy and also have more healthy behaviors [42]. This suggests that a positive view on future aging is important for healthy aging, as has been suggested by almost all women in our study, including the women without a healthy lifestyle or positive view themselves. Indeed, previous research demonstrated that older adults (65 years and older) who view their aging process as positive had a better functional health status in the future [43].

### Perspectives on determinants of healthy aging

Next to a positive view on aging, staying physically and mentally active were perceived as the most important determinants of healthy aging. Although this is in line with the well-known desire of older (migrant) adults to age in an active, engaged and healthy manner [7, 8, 44], specific perceived determinants remains relatively unstudied, especially among older women. A focus group study among older Palestinians (60 years and older) did demonstrate that having positive feelings, being socially engaged and having a good physical and mental health were perceived as factors that positively influenced healthy aging (i.e. determinants) [45]. This study, however, did not specifically report or analyze data for women and men separately.

### Differences between native-Dutch and Turkish-Dutch and Moroccan-Dutch older women

During our analyses, some consistent differences emerged between native-Dutch and Turkish-Dutch or Moroccan-Dutch women. First, religious and cultural beliefs played an important role in determining a healthy lifestyle regarding alcohol consumption and smoking behavior among Turkish-Dutch and Moroccan-Dutch older women. In contrast, native-Dutch women did not mention their religious beliefs as a motivator or barrier. In addition, with regard to their perspectives on determinants of healthy aging, Turkish-Dutch and Moroccan-Dutch women mentioned that aging is (also) in the hands of Allah, thereby describing a more limited reach of the influence of a healthy lifestyle, although recognized as important. Both differences are in line with previous findings demonstrating that religion based views influence the healthy lifestyle and perception of determinants of healthy aging among Moroccan-Dutch and Turkish-Dutch women aged 40 to 70 years [25, 37, 39].

When evaluating the results regarding migration background, it is important to note that they apply to first-generation immigrants. Over the coming years, second-generation immigrants will grow older and their beliefs and lifestyles might differ from their parents (and their native counterparts). Although literature on this topic is lacking, it seems that disadvantages in physical and psychological health at older ages remains among the first-generation Hispanic migrants in Italy and first-generation Turkish migrants in Germany, especially among women [46–48]. However, healthcare barriers have been suggested to decrease among second-generation Turkish immigrants in Germany [49], possibly influencing the motivators and barriers for a healthy lifestyle. Future research regarding this topic is advised.

### Implications for preventive healthy aging strategies

When exploring a preventive perspective on our results, there seems to be an opportunity for increasing physical activity to stimulate healthy aging among older women. Currently, about 30–40% of the older adults in the Netherlands meet the Dutch guidelines (moderate intensity physically active for at least two and half hours every week) [50, 51]. Our results demonstrate that older women have an intrinsic motivation to increase or maintain healthy physical activity, which is one of the most important success factors of behavior change [12]. Since the most important motivators were social peer pressure and personal preferences related to being outdoors, this confirms the effectiveness of group based physical activity interventions that take place outside among older women [52]. Since an additional barrier for being more physically activity was lack of time, intervention strategies should also focus on how to integrate these changes into these women’s daily schedule and daily routine. Specifically for Turkish-Dutch and Moroccan-Dutch older women, additional barriers were feeling tired and/or fear to get injured or overburden. Here, an additional focus on knowledge transfer regarding the benefits on energy levels and physical health of physical activity might be effective to include in the group-based activities. However, future research is needed to investigate this.

Our results also show room for increasing healthy diet behavior among older women and this was also regularly expressed as a wish for the future, also pinpointing an intrinsic motivation. Opportunities seem related to the social culture of unhealthy diet behavior and to the religious and cultural aspect among Turkish-Dutch and Moroccan-Dutch women specifically. This is in line with the results from a focus group study among native-Dutch, Turkish-Dutch and Moroccan-Dutch adults showing that social norms and practice make it difficult to adhere to a healthy diet [39] and an interview study among Turkish-Dutch and Moroccan-Dutch adult women demonstrating that cultural dishes prevents them from having a healthy diet [53]. Future research investigating this topic should focus on investigating ways to incorporate a healthy diet into daily social life and take into account the barrier “lack of time”, as demonstrated in previous studies [54]. For Turkish-Dutch and Moroccan-Dutch women this could mean investigating possibilities to combine healthy dishes with cultural dishes or replace some ingredients with healthier ones [53].

In addition, decreasing alcohol consumption among native Dutch older women also seems an opportunity for stimulating healthy aging. Alcohol consumption among Dutch older adults has increased over time, especially among women [50, 55]. Although women often mentioned the Dutch dietary guideline regarding alcohol consumption (“Don’t drink alcohol, if you do drink alcohol, no more than one glass a day.”) and consequent health benefit, we found a common consensus among native Dutch older women who regularly consume alcohol, that a little alcohol consumption (max one glass a day) had no negative health effects. This, however, has been recently proven not to be true by a large observational study [56]. Intervening on this knowledge gap may be included in strategies to lower alcohol consumption. However, a social cultural shift surrounding the cultural habit of alcohol consumption may have a larger impact since the main barriers to drink low alcohol are social related. However, such a shift takes time and requires additional (policy) measures, such as taxes and decreasing availability [57].

The possibility to actively improve sleep duration and quality is a rather unknown among the older women in our study. Although the importance of enough hours of sleep and good sleep quality for health and well-being among older adults has been increasingly recognized [58], this seems not reflected in the knowledge of the general older population. Even though older women more often have sleep problems and lower sleep quality compared to men [59]. To the best of our knowledge, studies investigating perspectives on and/or motivators and barriers for healthy sleep patterns among older adults are lacking.

### Strengths and limitations

Our study has several strengths and some limitations. The main strength of our study is to have investigated perspectives on five lifestyle factors in the Netherlands separately, allowing specific recommendations and insights. Furthermore, we successfully recruited older women with a broad range in age, educational levels and migration backgrounds, increasing the generalizablity of our study. It should be noted that there were less Turkish-Dutch and Moroccan-Dutch women than native Dutch women included in our study, although a high level of data saturation was reached. The digital nature of the interviews might have caused participation bias, since higher educated women are more likely to be able to use Zoom or digital interviews [60]. However, purpose sampling for lower educated older women, together with allowing phone interviews, enabled us to include them in our study [60]. More importantly, when analyzing the results, no clear differences emerged across educational levels. The digital nature of the interviews could have caused some bias in our results, since the surroundings and body language are harder to read, which can influence the depth and results of the interviews [60]. However, it could have also caused a more in depth and easy conversation, since it is held from their homes, which provides a comfortable situation [60]. Indeed, all participants were positive about the digital nature, although setting up could take some time. Another limitation that should be taken into account was that our participants had a relatively healthy lifestyle behavior compared to the Dutch general older women population. None of the participants were smokers, although the percentage of smokers among native Dutch older women (aged 55 years and older) in the general population was around 20% in 2018 [61]. Also, relatively more participants were higher educated (34% versus 21% in the general older Dutch women population), who in general have a more healthy lifestyle [62]. This might have biased our results to be less generalizable to the general older adult population in the Netherlands. It should also be noted that our study was performed during the corona pandemic, although we discussed motivators and barriers unrelated to the corona restrictions and all participants stated that the pandemic had not influenced their perspectives on determinants of healthy aging.

## Conclusion

Our results show that health was the most common motivator for all distinct lifestyles. Other motivators and barriers differed for distinct lifestyles and for Turkish-Dutch and Moroccan-Dutch women specific motivators and barriers were related to culture and religion. The largest opportunity to increase a healthy lifestyle and stimulate healthy aging among older women seems to be related to physical activity and healthy diet, due to the intrinsic motivation and wish to improve these. Furthermore, decreasing alcohol consumption among native-Dutch might also be beneficial for healthy aging among older native women. Our findings suggest that future research and prevention strategies aimed at improving lifestyle among older women should have a tailored, culture sensitive approach for distinct lifestyle factors.

## Data Availability

The data that supports the findings of the current study are not publicly available due to privacy regulations in accordance with the ethical committee of the Vrije Universiteit medical center (METC Amsterdam UMC, approval number 2020.0726) but are available from the corresponding author on reasonable request.
